# Reference-guided metagenomics reveals genome-level evidence of potential microbial transmission from the ISS environment to an astronaut's microbiome

**DOI:** 10.1016/j.isci.2021.102114

**Published:** 2021-01-29

**Authors:** Michael D. Lee, Aubrie O'Rourke, Hernan Lorenzi, Brad M. Bebout, Chris L. Dupont, R. Craig Everroad

**Affiliations:** 1 Exobiology Branch, NASA Ames Research Center, Mountain View, CA, USA; 2 Blue Marble Space Institute of Science, Seattle, WA, USA; 3 Space Life Sciences, NASA Kennedy Space Center, Merritt Island, FL, USA; 4 Department of Infectious Diseases, J. Craig Venter Institute, Rockville, MD, USA; 5 J. Craig Venter Institute, San Diego, CA, USA

**Keywords:** Microbiology, Space Science

## Abstract

Monitoring microbial communities aboard the International Space Station (ISS) is essential to maintaining astronaut health and the integrity of life-support systems. Using assembled genomes of ISS-derived microbial isolates as references, recruiting metagenomic reads from an astronaut's nasal microbiome revealed no recruitment to a *Staphylococcus aureus* isolate from samples before launch, yet systematic recruitment across the genome when sampled after 3 months aboard the ISS, with a median percent identity of 100%. This suggests that either a highly similar *S*. *aureus* population colonized the astronaut's nasal microbiome while the astronaut was aboard the ISS or that it may have been below detection before spaceflight, instead supporting a shift in community composition. This work highlights the value in generating genomic libraries of microbes from built-environments such as the ISS and demonstrates one way such data can be integrated with metagenomics to facilitate the tracking and monitoring of astronaut microbiomes and health.

## Highlights

• Understanding built-environment microbiomes is critical for human space exploration
• Reference-guided metagenomics is a powerful approach for monitoring microbiomes
• We show potential microbial colonization of an astronaut microbiome while aboard the ISS

## Graphical Abstract

**Figure:**
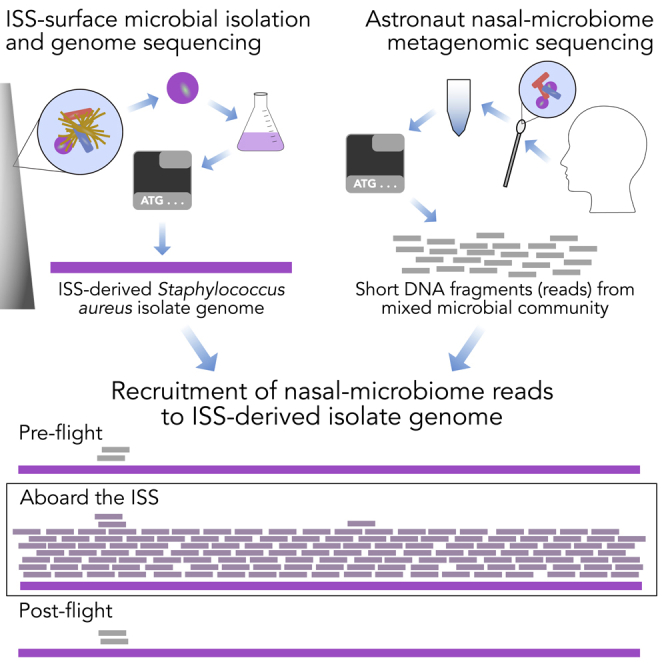

## Introduction

There is a growing awareness and consideration as to how the microbiome of built-environments affects human health (e.g., National Academies of Sciences and Medicine, 2017). The International Space Station (ISS) represents a particularly unique built-environment given its physical isolation, relatively low rate of occupant quantity, and turnover and impacts on immunity and health in general due to environmental factors such as microgravity and radiation. Tracking and monitoring of microbial communities within space-based built-environments such as the ISS is essential to maintaining astronaut health and preserving the integrity of life-support components such as potable-water and food-production systems. Particularly as we begin to look toward longer-duration human-spaceflight missions (Voorhies and Lorenzi, 2016), we will need to continue, and expand, our practices in gathering and utilizing information on the microbial populations inhabiting these closed environments that are traveling with us.

Microbial surveillance of the ISS environment by NASA has been underway in different respects for some time. These efforts have included culture-based approaches that have allowed the characterization of isolates recovered from surfaces (e.g., Knox et al., 2016; Sielaff et al., 2017; Romsdahl et al., 2018; Blachowicz et al., 2019) and the ISS potable-water system (O'Rourke et al., 2020). Culture-independent methods have also been employed, including targeted-amplicon sequencing (e.g., 16S ribosomal RNA, e.g., Checinska et al., 2015; Sielaff et al., 2019; Voorhies et al., 2019) and metagenomic sequencing (e.g., Be et al., 2017; Singh et al., 2018; Avila-Herrera et al., 2020) of surfaces and particles. Some of the primary findings from these have included demonstrating that the ISS microbiome differs from spacecraft-assembly clean rooms (Be et al., 2017), that different ISS surface areas appear to harbor different microbiomes (Sielaff et al., 2019), and that human skin-associated microorganisms seem to be a primary source for the microbiomes of ISS surfaces (Checinska et al., 2015; Voorhies et al., 2019). A few studies incorporating targeted-amplicon sequencing focusing on astronaut microbiomes have also been published. A study on astronaut salivary microbiomes revealed an increase in alpha-diversity during spaceflight (Urbaniak et al., 2020). Additional work focusing on different astronaut microbiome sources detected varied alpha-diversity responses during spaceflight for feces (increased), skin (mixed), and nasal (decreased) microbiomes, in addition to identifying consistent trends such as an overall decrease in Gammaproteobacteria sequences recovered from skin samples and an overall increase in *Staphylococcus* spp. in nasal samples (Voorhies et al., 2019).

The current work combines newly sequenced and assembled genomes of *Staphylococcus* microbial isolates recovered from the ISS with astronaut nasal microbiome metagenomic data sampled before, during, and after their time aboard the ISS. This is an observational, *ex post facto* integration and exploration of these datasets demonstrating one avenue of leveraging different aspects of NASA's efforts toward tracking microbial communities aboard the ISS.

## Results and discussion

Fifty-three *Staphylococcus* isolates were recovered from ISS surfaces between 2006 and 2015, sequenced, and their genomes assembled (see Transparent Methods). These included 47 *Staphylococcus epidermidis*, 3 *S*. *aureus* (all lacking the *mecA* gene indicative of methicillin resistance; Wielders et al., 2002), and 1 each of *Staphylococcus auricularis*, *Staphylococcus lugdunensis*, and *Staphylococcus saprophyticus* as classified by NCBI (Figure S1; Table S1). Metagenomic sequencing was performed on DNA extracted from nasal swabs collected from 5 astronauts at multiple time points before (n = 3 or 4), during (n = 3), and after (n = 3) their time aboard the ISS for 6-month missions (Voorhies et al., 2019; Tables S2; S3).

Although we have the dates of isolation for the isolates, information is not publicly available for dates on specific astronaut missions, leaving us only able to discuss the metagenomic microbiome datasets in terms of relative time (i.e., days before, during, and after flight). Following removal of human reads, the average number of reads per metagenomic sample was 766,906 ± 421,561 (mean ± 1 SD; Table 1), which incorporating the read size equates to ∼115 ± 63.2 Mbp. This relatively low coverage precludes doing extensive analyses on any microbial members present (see limitations of the study section), but as presented later can still enable presence or absence detection at the genome level.

**Table 1:** Overview of metagenomic samples

Subject ID	# Samples	# Reads∗ per sample (mean ±1 SD)	Megabases (mean ±1 SD)
AstB	10	448,828 ± 60,542	67.3 ± 9.08
AstC	9	786,374 ± 559,220	118 ± 83.9
AstE	9	1,110,925 ± 567,810	167 ± 85.2
AstG	9	828,184 ± 251,201	124 ± 37.7
AstH	10	702,696 ± 210,988	105 ± 31.6

∗Count is of total reads remaining after human-read removal.

We dereplicated the genomes (i.e., chose single representatives for clusters of highly similar genomes) before recruiting the nasal microbiome metagenomic reads (see Transparent Methods). This reduced the total number of ISS-derived reference genomes being used to recruit to from 53 to 7 (Table S4). Read-mapping was carried out for each metagenomic sample to each of the seven dereplicated isolate genomes (Table S5). A minimum detection (proportion of the genome that recruited reads) of at least 20% was employed to filter out sample-to-isolate mappings that were potentially due to non-specific read-recruitment. Two *S*. *epidermidis* isolates (s29 and s32) and two *S*. *aureus* isolates (s9 and s42) surpassed this threshold in at least 1 sample in all 5 astronaut datasets (Table S5).

### *S. epidermidis* read-recruitment was relatively consistent across all samples

**Figure 1:**
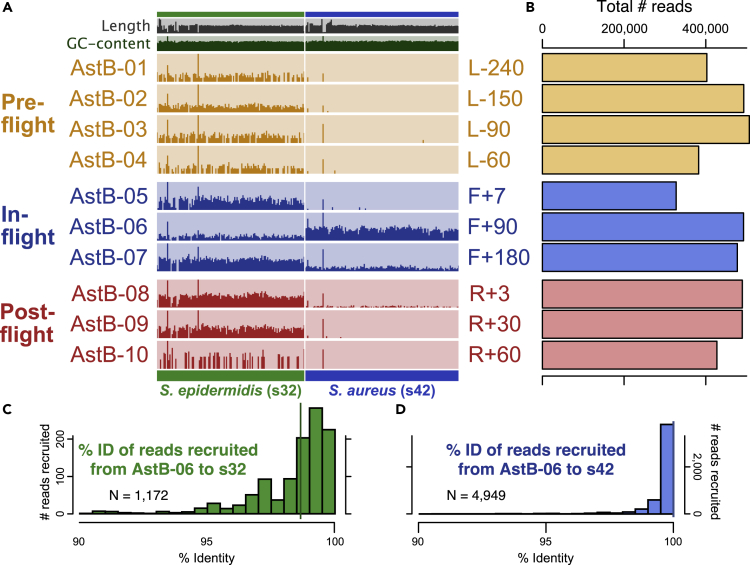
Visualization of metagenomic read-recruitment from astronaut AstB's nasal microbiome through time to genomes of two microbial isolates recovered from surfaces of the ISS (A) Stretched across the x axis at the bottom are 2 genomes of microbial isolates recovered from the ISS, and the small, vertical columns above them represent information about ~20,000-bp fragments of the genome. The top 2 rows depict length and GC-content of those fragments across the genomes. Following those, each row represents a different time point of astronaut AstB's nasal microbiome, with metagenomic read-recruitment to the ISS-derived isolate genome presented with each fragment's mean coverage normalized to the overall mean coverage for that sample. The green *S*. *epidermidis* on the left represents what consistent read recruitment from almost all time points looks like. In contrast, reads are recruited consistently across genome *S*. *aureus* s42 (blue, on right) after 90 and 180 days aboard the ISS, and 3 days after returning to Earth, but not at any other time points. Lone high recruitment peaks are due to highly conserved regions beyond *Staphylococcus* (see Transparent Methods, “metagenomic read-recruitment, analysis, and visualization). (B) Depicts the total number of reads available from each sample following human-read removal. (C and D) Histograms depicting the percent identities of the reads recruited from AstB-06 to the s32 (median percent ID of recruited reads of 98.68%) and s42 (median percent ID of 100%) genomes. Vertical bars represent medians.

All 5 astronaut datasets recruited to the s29 and s32 *S*. *epidermidis* reference genomes relatively consistently across all samples (e.g., Figure 1A and s32 *S*. *epidermidis* on left side; Figure S2; Table S5). Read-mapping necessarily allows for some variation, however, and based upon the percent identities of the reads recruited to the isolate genomes, all datasets held *S*. *epidermidis* populations that were more similar to the s32 reference (median percent identity of 98.68%) than the s29 reference (97.97%; Welch t test p = 6 × 10^−4^). With the most similar ISS-derived isolate genome being around 98.7% identical to the reads recovered from the astronauts before, during, and after their time aboard the ISS, this serves as an example of consistently present *S*. *epidermidis* populations in all astronaut nasal microbiomes that are simply similar enough to recruit reads to our reference genomes.

### *S. aureus* read-recruitment was exclusive to AstB and AstE

*S*. *aureus* ISS-derived genomes were only detected in the nasal microbiome datasets AstB and AstE (Table S5). AstE recruited reads across both s9 and s42 *S*. *aureus* genomes for all time points before, during, and after flight with about the same percent identity (median of 98.67% identity to each; Figures S3–S5; Table S5). As with the aforementioned consistent *S*. *epidermidis* recruitment, this reveals that the AstE nasal microbiome held a population of *S*. *aureus* before, during, and after flight that was similar to the ISS-derived *S*. *aureus* genomes, and we do not see any change during their time aboard the ISS. For AstB, on the other hand, *S*. *aureus* ISS-derived genomes were only detected during their time aboard the ISS.

### AstB recruits systematically to *S. aureus* In-Flight only

Recruitment of metagenomic data from AstB's nasal microbiome through time revealed no recruitment to *S*. *aureus* in 5 samples spanning from 240 days before launch up to being aboard the ISS for 7 days. However, there was systematic read-recruitment across the entire *S*. *aureus* s9 and s42 genome when sampled at 90 and 180 days aboard the ISS that remained detectable at 3 days following the astronaut's return to Earth (R+3), but not at 30 or 60 days after returning to Earth (Figure 1). This suggests either that the astronaut's nasal microbiome was colonized by a population similar to the ISS-derived *S*. *aureus* isolates while the astronaut was aboard the ISS, followed by subsequent carriage back to Earth, or that its relative abundance was below detection before and after spaceflight.

### AstB's In-Flight *S. aureus* population is 100% identical to the ISS-derived isolate s42 *S. aureus* genome

**Figure 2:**
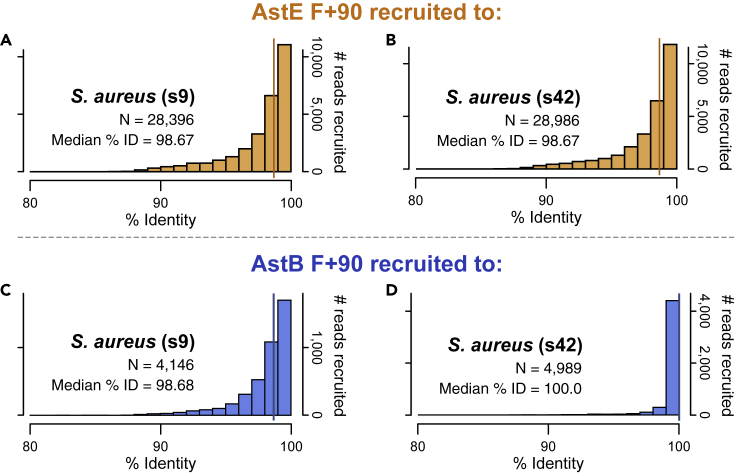
Histograms of percent identities of reads recruited to ISS-derived *S*. *aureus* isolate genomes (A–D) (A and B) AstE's In-Flight day-90 nasal metagenome reads recruited to the 2 *S*. *aureus* isolates, showing a median percent identity of 98.67% to both. (C and D) AstB's same time point recruited to the same 2 *S*. *aureus* isolate genomes as (A and B), showing that unlike the population in AstE's nasal microbiome, the recruited reads have a median percent identity of 100% to one of the isolate references, *S*. *aureus* s42 (D).

In the case of all nasal microbiomes recruiting to *S*. *epidermidis*, and all of AstE's samples' reads recruiting to *S*. *aureus* isolates, the microbial populations that were consistently present before, during, and after flight were about 98.5% similar to the ISS-derived isolate genomes based on percent identity of recruited reads (Figures S2–S5; Tables S5 and S6). Figures 2A and 2B depict what this looks like for AstE's 90-day In-Flight time point (“F+90”), where the percent identity of the reads recovered from the astronaut's nasal *S*. *aureus* population has a long-tail distribution and median of 98.67% to both *S*. *aureus* ISS-derived isolate genomes s9 (Figure 2A) and s42 (Figure 2B)—the same is true for all AstE's time points (Figures S4 and S5; Tables S5 and S6). This type of recruitment can be thought of as “off-target” in the sense that there were indeed microbial populations of *S*. *aureus* present, but the reads recovered from them were only roughly 98.5% identical to the ISS-derived isolate genomes. This can also be seen for the recruitment of AstB to ISS-derived *S*. *aureus* s9, having a median of 98.7% ID of reads recruited and a similar long-tail distribution (Figure 2C). However, the same sample recruited to ISS-derived *S*. *aureus* s42 shows the reads recovered have a median percent identity of 100%, without the long-tail distribution (Figure 2D). Based on a phylogenomic tree with NCBI RefSeq complete genomes built with 119 single-copy genes specific to the Firmicutes phylum (Lee, 2019; Data S1), s42 is most closely related to *S*. *aureus* strain CFSAN082783 (GCF_008330585.1), and the average nucleotide identity between the two is 99.8% (Jain et al., 2018). Multi-locus sequence-typing based on the seven genes in the PubMLST (Jolley and Maiden, 2010) *S*. *aureus* database categorized both the nearest relative strain, CFSAN082783, and ISS-derived isolate s42 as sequence-type (ST) 45, clonal complex 45.

### Conclusion

Here we present evidence at the genomic level of either colonization or enrichment of a microbial population at of *S*. *aureus* in the nasal microbiome of an astronaut while aboard the ISS—a population that based on read-recruitment is virtually identical to an ISS-derived isolate. These results build on our previously reported amplicon-based characterization of these same nasal microbiome samples that noted an increase in recovered *Staphylococcus* sequences (Voorhies et al., 2019). Upper respiratory symptoms including congestion, rhinitis, and sneezing were some of the most reported incidences in a study of astronauts from 46 long-duration ISS missions (Crucian et al., 2016), and greater abundances of *S*. *aureus* in the upper respiratory tract have been shown to be associated with several respiratory diseases (e.g., Feazel et al., 2012; Muluk et al., 2018; Ramakrishnan et al., 2013). It is plausible that the increased relative abundances of *S*. *aureus* in the nasal microbiomes of astronauts while aboard the ISS is a factor in causing some of these symptoms and is worth investigating further.

This is the first report demonstrating colonization or enrichment of an ISS microbial population at the genomic level within an astronaut's nasal microbiome. The lack of detection of the population at 30 days after returning to Earth suggests that this may have been a transient event, although we cannot rule out it may have simply been below detection. This work highlights the value in generating genomic libraries of microbes from built-environments such as the ISS and demonstrates one way that genomic and metagenomic data can be integrated to facilitate efforts to track and monitor astronaut microbiomes—efforts that will become increasingly important as we begin undertaking longer-duration human-spaceflight missions.

### Limitations of the study

The average number of metagenomic reads per sample following removal of human-derived reads was 766,906 ± 421,561 (mean ± 1 SD; Table 1). Incorporating read-size this equated to ∼115 ± 63.2 Mbp of possible coverage per sample. To put this in context of thinking about a mixed microbial community, even if there were only one microbial organism with a 5-Mbp genome present in the sampled microbiomes, this would only leave just over ∼20× coverage for that one genome. Although genome-level presence/absence detection was still attainable as presented earlier, this relatively low coverage precluded the ability to do extensive analyses on any microbial members present.

### Resource availability

#### Lead contact

Further information should be directed to and will be fulfilled by the Lead Contact, Michael D. Lee (Mike.Lee@nasa.gov).

#### Materials availability

This study did not generate new unique reagents.

#### Data and code availability

Due to IRB considerations, the Astronaut metagenomic data is available upon request from NASA's Life Sciences Data Archive (LSDA) through experiment 1836 (lsda.jsc.nasa.gov/Experiment/exper/1836). The ISS-derived *Staphylococcus* isolate genomes are available through NCBI at BioProject: PRJNA486830 (Table S1) and at our Open-Science Framework repository (OSF; Foster and Deardorff, 2017), project “mr582,” which also holds walkthroughs and annotated code for the processing and analyses that were performed (see osf.io/mr582/wiki/).

## Methods

All methods can be found in the accompanying Transparent methods supplemental file.

## Supplemental information

Document S1. Transparent methods and Figures S1–S5

Table S1. Isolate assembly information

Table S2. Metagenome samples recovered

Table S3. Metagenome information

Table S4. Isolate assembly dereplication information

Table S5. Read-mapping summary information

Table S6. Recruited reads percent ID table

Data S1. Phylogenomic tree of *S. aureus* in Newick format
