# Supplementary material for: Reference-guided metagenomics reveals genome-level evidence of potential microbial transmission from the ISS environment to an astronaut's microbiome
Source: iScience. 2021 Jan 29;24(2):102114. doi: 10.1016/j.isci.2021.102114 (PMC7892915; doi:10.1016/j.isci.2021.102114)
Supplement: Document S1. Transparent methods and Figures S1–S5 [file mmc1.pdf]

iScience, Volume *24*

Supplemental Information

Reference-guided metagenomics reveals genome-level evidence of potential microbial transmission from the ISS environment to an astronaut's microbiome

Michael D. Lee, Aubrie O'Rourke, Hernan Lorenzi, Brad M. Bebout, Chris L. Dupont, and R. Craig Everroad

Supplemental Data Items

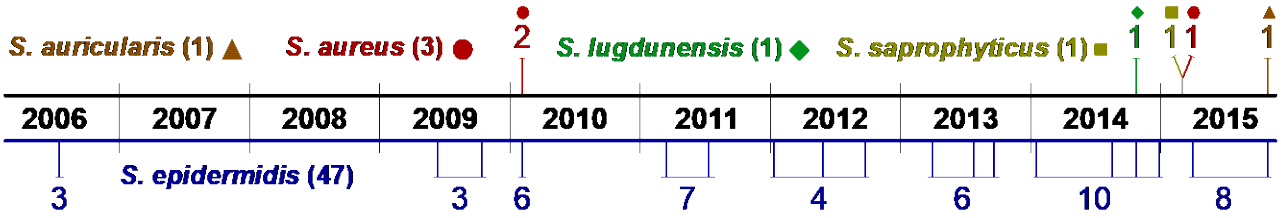

Figure S1. Isolation timeline, Related to first paragraph of Results and Discussion, and Table S1. The 3 *S. aureus* isolates were isolated only at one sampling timepoint in 2010 (2 of them, s8 and s9), and then one timepoint in 2015 (s42). Data in Table S1.

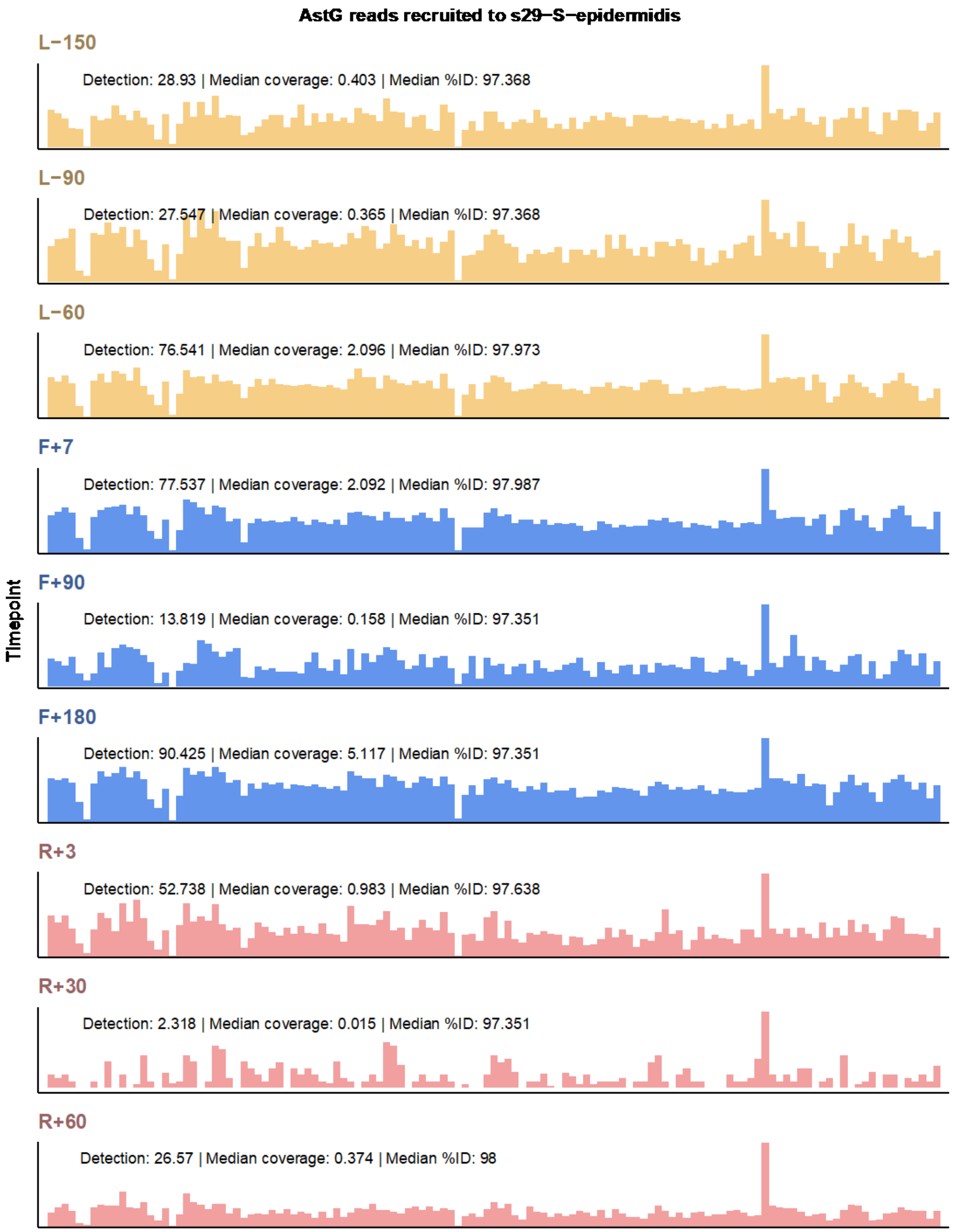

Figure S2. Read recruitment from all AstG timepoints to *S. epidermidis* s29 isolate genome, Related to Figure 1A. Bars represent 20,000 bp fragments of the genome, so the whole genome spans the x-axis. Proportion of the genome covered ("Detection"), median coverage, and median percent ID of all aligned reads based on BLAST are overlain in text on each. All 5 nasal microbiome datasets looked similar (Table S5).

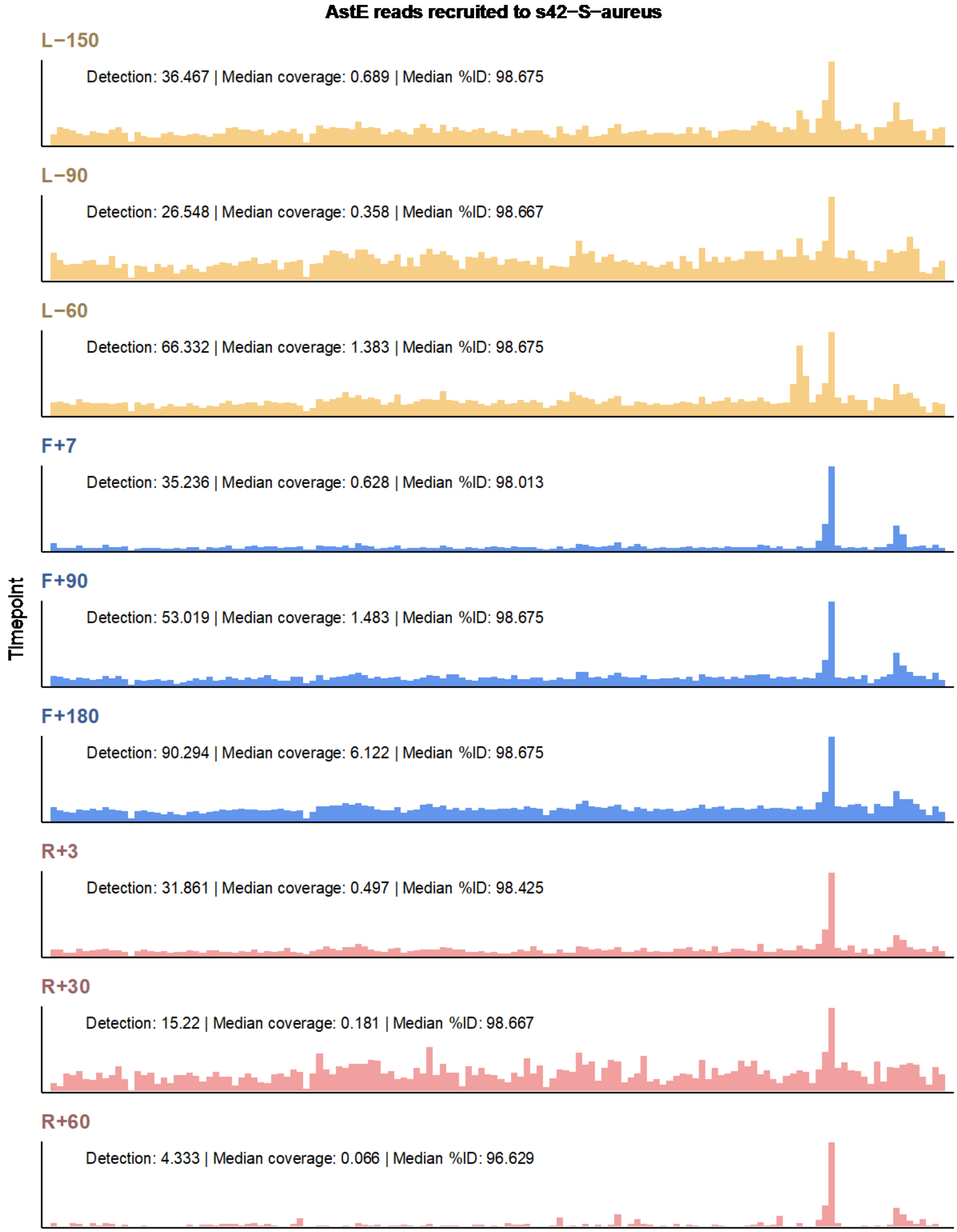

Figure S3. Read recruitment from all AstE timepoints to *S. aureus* s42 isolate genome, Related to sections '*S. aureus read-recruitment was exclusive to AstB and AstE*' and '*AstB's In-Flight S. aureus population is 100% identical to the ISS-derived isolate s42 S. aureus genome*'.Bars represent 20,000 bp fragments of the genome, so the whole genome spans the x-axis. Proportion of the genome covered ("Detection"), median coverage, and median percent ID of all aligned reads based on BLAST are overlain in text on each. Recruitment of AstE to *S. aureus* s9 looked similar (Table S6).

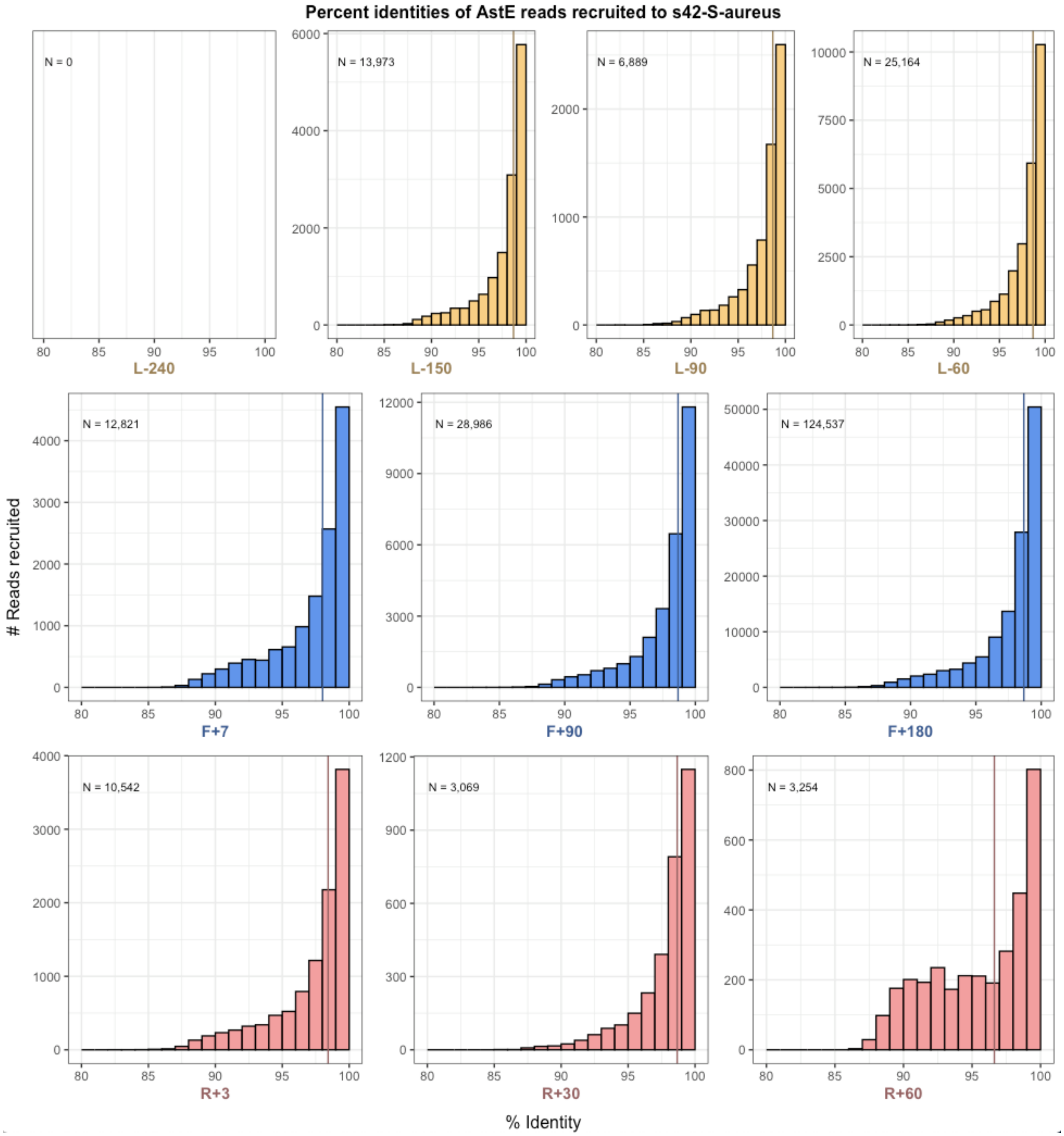

Figure S4. Percent identities of reads recruited from all AstE timepoints to *S. aureus* s42 isolate genome, Related to sections '*S. aureus read-recruitment was exclusive to AstB and AstE'* and '*AstB's In-Flight S. aureus population is 100% identical to the ISS-derived isolate s42 S. aureus genome'*. Percent ID is based on BLAST of aligned reads to the genome. AstE to *S. aureus* s9 looked similar (Figure S5; Tables S5 and S6).

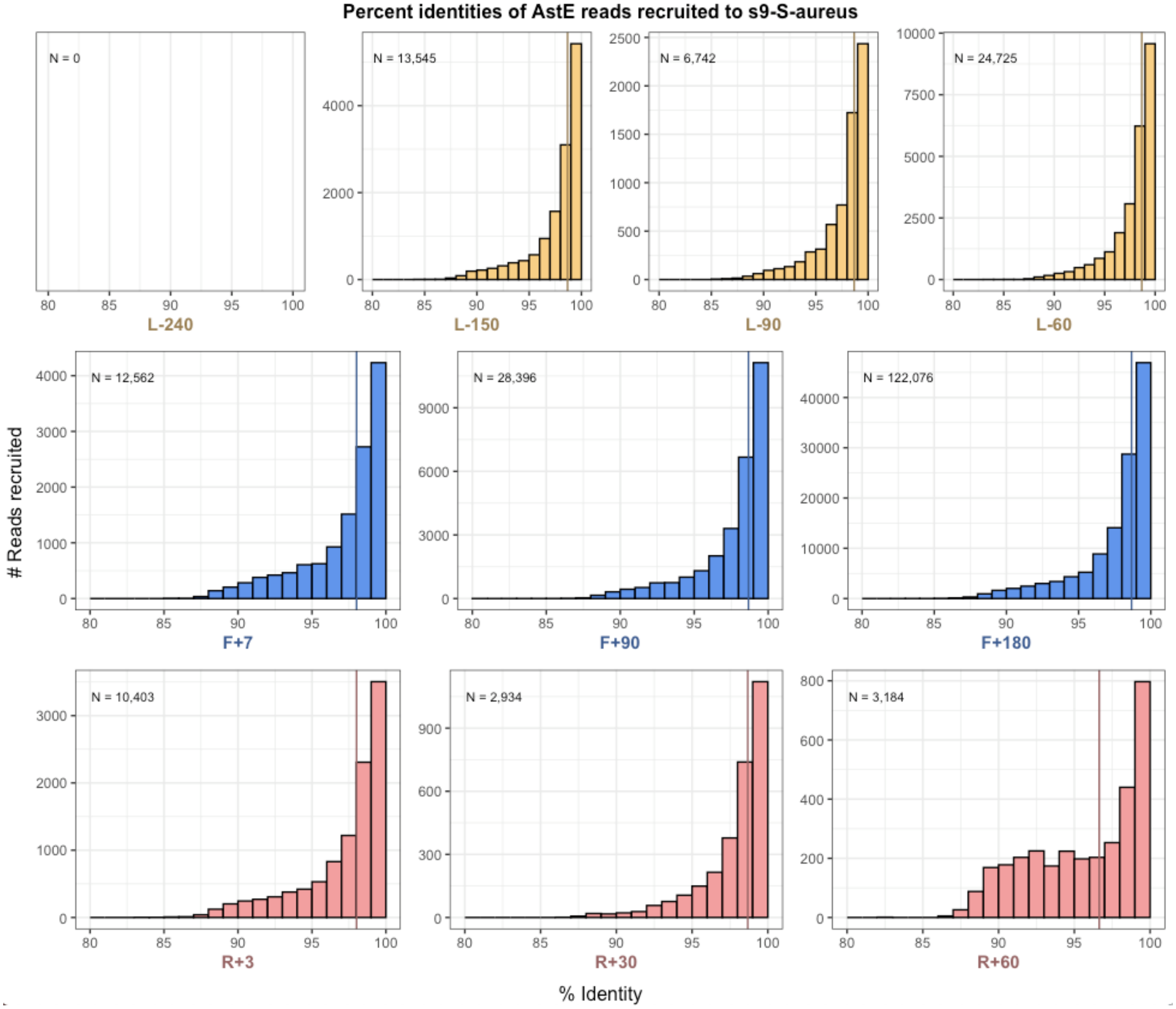

Figure S5. Percent identities of reads recruited from all AstE timepoints to *S. aureus* s9 isolate genome. '*AstB's In-Flight S. aureus population is 100% identical to the ISS-derived isolate s42 S. aureus genome'*. Percent ID is based on BLAST of aligned reads to the genome. AstE to *S. aureus* s42 looked similar (Figure S4; Tables S5 and S6).

Transparent Methods

Organism isolation

Staphylococcus isolates were collected from surfaces of the ISS using the Surface Sampler Kit (https://lsda.jsc.nasa.gov/Hardware/hardw/670) between 2006–2015, isolated on tryptic soy agar (TSA) plates (see Figure S1 and Table S1 for dates of isolation), and stored at -80°C in 15% glycerol at NASA's Johnson Space Center – independent of the current study – as part of NASA's long-term efforts to recover microbial isolates from the ISS (see LSDA experiment 13823; https://lsda.jsc.nasa.gov/Experiment/exper/13823). The authors of the current study (specifically MDL and AO) received these isolates as slant cultures that were sent to the J. Craig Venter Institute (JCVI) in San Diego, California, USA. Isolates were plated on TSA places, incubated overnight at 35°C, and inoculated into 4ml of tryptic soy broth. 2ml were centrifuged to form pellets which were frozen at -80°C until DNA extractions were performed.

Nasal microbiome sampling

The astronaut sampling strategy is originally detailed in Voorhies et al., 2019, and is recapitulated here. Longitudinal samples were collected from 5 astronauts (indicated as AstB, AstC, AstE, AstG, AstH) at 9 or 10 timepoints (Table S2). Sample timepoints are reported as related to: 1) the astronaut's launch date to the ISS (being indicated by "L-" and the number of days prior to launch); 2) the astronaut's time aboard the ISS during their mission (being indicated by "F+" and the number of days aboard the ISS); and 3) the astronaut's return to Earth (being indicated by "R+" and the number of days after returning). Samples were self-collected by the astronauts via nasal swabs which were stored at -100°C aboard the ISS until returned to Earth.

DNA extraction

DNA was extracted from isolates using a phenol/chloroform protocol and quantified using a nanodrop and run on a gel to inspect quality. For the metagenomes, as described in Voorhies *et al.*, 2019, after sample collection, nasal swabs were resuspended in microcentrifuge tubes containing 1.2ml lysis buffer (20mM Tris-Cl, pH 8, 2mM EDTA, 1.2% Triton X-100). Following vortexing at maximum speed for one minute, 1ml of solution was removed and placed in a lysing Matrix B tube (MP Biomedicals CAT # 6911-500). These were vortexed at maximum speed for 5 minutes, then at 10,000 RPM for 1 minute. 700 µl of solution was then incubated for ten minutes at 75°C, allowed to cool, treated with 200mg/ml lysozyme and 20mg/ml Proteinase K, and then phenol/chloroform extractions were performed.

DNA library preparation and sequencing

Microbial-isolate DNA library preparation was performed with the NEBNext Ultra II FS DNA Library Prep Kit for Illumina (New England Biolabs) following the manufacturer's instructions, but using half the standard reaction volumes. Metagenomic libraries were prepared with the NexteraXT DNA Library Prep Kit (Illumina), following the manufacturer's specifications. Sequencing for both isolates and metagenomes was performed on Illumina's NextSeq 500 platform with 2x150 paired-end sequencing targeting roughly 1GB of data per sample. Human reads were removed from the metagenomic data using BMTagger (srprism v2.3.17; bmtool v0.0.0; ftp://ftp.ncbi.nlm.nih.gov/pub/agarwala/bmtagger/) with human assembly HG18 as the reference.

Isolate sequencing-data preprocessing, assembly, and dereplication

For all isolate sequence datasets, adapters were searched and removed with bcl2fastq2 Conversion Software v2.17 (Illumina). Quality profiles of read pairs were generated with FastQC v0.11.8 (Andrews, 2010), and trimming was performed with Trimmomatic v0.39 (Bolger et al., 2014) using a sliding window setting of 5:20 and filtering out reads shorter than 100 bp after trimming. Assemblies were performed using SPAdes v3.12.0 (Bankevich et al., 2012) with default settings. Assemblies were summarized with bit v1.8.06 (Lee, 2018) and estimated completion and redundancy were calculated with checkm v1.1.2 (Parks et al., 2015). Assembly summary information is presented in Table S1. The mecA gene was searched in *S. aureus* isolates via BLASTp (Altschul et al., 1990) using UniProtKB (Consortium, 2017) protein Q7DHH4. Isolate assemblies were dereplicated with dRep v2.6.2 (Olm et al., 2017) prior to initial mapping.

Metagenomic read-recruitment, analysis, and visualization

Metagenomic reads were recruited to individual isolate genomes with bowtie2 v2.4.1 (Langmead and Salzberg, 2012) with default settings. Sam files were converted to bam files and sorted and indexed with samtools v1.7 (Li et al., 2009), and bedtools v2.29.2 (Quinlan and Hall, 2010) was utilized to help calculate detection (portion of reference that recruited reads). Reads that successfully mapped were then aligned with

BLAST v2.5.0 (Altschul et al., 1990). Snakemake v5.19.1 (Köster and Rahmann, 2012) was used for much of the processing performed (see OSF osf.io/mr582/wiki/). Analyses were performed and some visualizations generated using R v3.6.3 (R Core Team, 2017) in RStudio v1.1.456 (RStudio Team, 2020), making heavy use of the tidyverse v1.3.0 (Wickham et al., 2019) and ggpubr v0.4.0 (Kassambara, 2020) packages. Read-recruitment across the genomes was also visualized with anvi'o v6.2 (Eren et al., 2015). For Figure 1, read-mapping was performed to a combined index of both *S. epidermidis* s32 and *S. aureus* s42.

The large peak in *S. aureus* s42 in Figure 1A is due to non-specific read recruitment to a highly conserved DNA sequence. The sequence responsible for its recruitment is:

```
>identical_sequence_between_s32_and_s42
AGCTATGCCGTTGGCACGACAACTGGTACACCAGAGGTATGTCCATCCCGGTCCTCTCGTACTAAGGAC
AGCTCCTCTCAAATTTCCTACGCCCACGACGGATAGGGACCGAACTGTCTCACGACGTTCTGAACCCAG
CTCGCGTACCGCTTTAATGGGCGAACAGCCCAACCCTTGGGACCGACTACAGCCCCAGGATGCGATGA
GCCGACATCGAGGTGCCAAACCTCCCCGTCGATGTGAACTCTTGGGGGAGATAAGCCTGTTATCCCCG
GGGTAGCTTTTATCCGTTGAGCGATGGCCCTTCCATGCGGAACCACCGGATCACTAAGTCCGTCTTTCG
ACCCTGCTCGACTTGTAGGTCTCGCAGTCAAGCTCCCTTATGCCTTTACACTCTATGAATGATTTCCAAC
CATTCTGAGGGAACCTTTGAGCGCCTCCGTTACCTTTTAGGAGGCGACCGCCCCAGTCAAACTGCCCG
CCTGACACTGTCTCCCACC
```

This is identical to the central high peak of read recruitment seen in *S. epidermidis* s32 also. A BLASTN search yields many *Staphylococcus* with 100% identical, complete alignments, and if excluding the *Staphylococcus* genus, there are many different organisms with near 100% identity and full alignments. A BLASTX search yields hypothetical, uncharacterized, and conserved proteins, RNAases, and cell-wall associated hydrolases from many different organisms.

Multi-locus sequence-typing

mlst v2.19.0 (Seemann) was utilized to perform multi-locus sequence-typing of the *S. aureus* assemblies based on PubMLST (Jolley and Maiden, 2010).

Phylogenomic tree construction and average nucleotide identity calculation

GToTree v1.5.36 (Lee, 2019) was utilized to make a phylogenomic tree of ISS-derived isolates and those available through NCBI's RefSeq (Leary et al., 2016) using a single-copy gene-set of 119 target genes specific for bacteria of the phylum Firmicutes. GToTree by default downloads all reference genomes, identifies coding sequences as needed in all input genomes with prodigal v2.6.3 (Hyatt et al., 2012), scans amino-acid sequences for the target genes with HMMER3 v3.3.1 (Eddy, 2011), aligns each gene-set with Muscle v3.8.1551 (Edgar, 2004), trims the alignments with trimal v1.4.rev15 (Capella-Gutiérrez et al., 2009), concatenates all gene-set alignments, swaps labels for taxonomic information with TaxonKit v0.6.0 (Shen and Xiong, 2019), and then uses FastTree2 v2.1.10 (Price et al., 2010) for phylogenetic estimation. Average nucleotide identity was calculated with fastANI v1.32 (Jain et al., 2018).

General

Conda (Anaconda-Team, 2016) and Snakemake (Köster and Rahmann, 2012) were utilized throughout this work.

Data and code availability

Due to IRB considerations, the Astronaut metagenomic data is available upon request from NASA's Life Sciences Data Archive (LSDA) through experiment 1836 (lsda.jsc.nasa.gov/Experiment/exper/1836). The ISS-derived *Staphylococcus* isolate genomes are available through NCBI under project accession PRJNA486830 (Table S1) and at our Open-Science Framework repository (OSF; Foster and Deardorff, 2017), project "mr582", which also holds walkthroughs and annotated code for the processing and analyses that were performed (see osf.io/mr582/wiki/).

Supplemental References

Altschul, S.F., Gish, W., Miller, W., Myers, E.W., and Lipman, D.J. (1990). Basic local alignment search tool.

J. Mol. Biol. *215*, 403–410.

Anaconda-Team (2016). Anaconda Software Distribution.

Andrews, S. (2010). FastQC: A Quality Control Tool for High Throughput Sequence Data.

Bankevich, A., Nurk, S., Antipov, D., Gurevich, A.A., Dvorkin, M., Kulikov, A.S., Lesin, V.M., Nikolenko, S.I., Pham, S., Prjibelski, A.D., et al. (2012). SPAdes: A new genome assembly algorithm and its applications to single-cell sequencing. J. Comput. Biol. *19*, 455–477.

Bolger, A.M., Lohse, M., and Usadel, B. (2014). Trimmomatic: A flexible trimmer for Illumina sequence data. Bioinformatics *30*, 2114–2120.

Capella-Gutiérrez, S., Silla-Martínez, J.M., and Gabaldón, T. (2009). trimAl: A tool for automated alignment trimming in large-scale phylogenetic analyses. Bioinformatics *25*, 1972–1973.

Consortium, T.U. (2017). UniProt : the universal protein knowledgebase. *45*, 158–169.

Eddy, S.R. (2011). Accelerated profile HMM searches. PLoS Comput. Biol. *7*.

Edgar, R.C. (2004). MUSCLE: A multiple sequence alignment method with reduced time and space complexity. BMC Bioinformatics *5*, 1–19.

Eren, A.M., Esen, C., Quince, C., Vineis, J.H., Morrison, H.G., Sogin, M.L., and Delmont, T.O. (2015). Anvi ' o : an advanced analysis and visualization platform for ' omics data. 1–29.

Foster, E.D., and Deardorff, A. (2017). Open Science Framework (OSF). J. Med. Libr. Assoc. *105*, 203–206.

Hyatt, D., Locascio, P.F., Hauser, L.J., and Uberbacher, E.C. (2012). Gene and translation initiation site prediction in metagenomic sequences. Bioinformatics *28*, 2223–2230.

Jain, C., Rodriguez-R, L.M., Phillippy, A.M., Konstantinidis, K.T., and Aluru, S. (2018). High throughput ANI analysis of 90K prokaryotic genomes reveals clear species boundaries. Nat. Commun. *9*, 1–8.

Jolley, K.A., and Maiden, M.C.J. (2010). BIGSdb: Scalable analysis of bacterial genome variation at the population level. BMC Bioinformatics *11*, 595.

Kassambara, A. (2020). ggpubr: “ggplot2” based publication-ready plots.

Köster, J., and Rahmann, S. (2012). Snakemake—a scalable bioinformatics workflow engine. Bioinformatics *28*, 2520–2522.

Langmead, B., and Salzberg, S.L. (2012). Fast gapped-read alignment with Bowtie 2. Nat. Methods *9*, 357–359.

Leary, N.A.O., Wright, M.W., Brister, J.R., Ciufo, S., Haddad, D., Mcveigh, R., Rajput, B., Robbertse, B., Smith-white, B., Ako-adjei, D., et al. (2016). Reference sequence (RefSeq) database at NCBI : current status , taxonomic expansion , and functional annotation. *44*, 733–745.

Lee, M.D. (2018). Bioinformatics Tools (bit). doi:10.5281/zenodo.3383647

Lee, M.D. (2019). GToTree: a user-friendly workflow for phylogenomics. Bioinformatics 1–3.

Li, H., Handsaker, B., Wysoker, A., Fennell, T., Ruan, J., Homer, N., Marth, G., Abecasis, G., and Durbin, R. (2009). The Sequence Alignment/Map format and SAMtools. Bioinformatics *25*, 2078–2079.

Olm, M.R., Brown, C.T., Brooks, B., and Banfield, J.F. (2017). DRep: A tool for fast and accurate genomic comparisons that enables improved genome recovery from metagenomes through de-replication. ISME J. *11*, 2864–2868.

Parks, D.H., Imelfort, M., Skennerton, C.T., Hugenholtz, P., and Tyson, G.W. (2015). CheckM : assessing the quality of microbial genomes recovered from isolates , single cells , and metagenomes. 1043–1055.

Price, M.N., Dehal, P.S., and Arkin, A.P. (2010). FastTree 2 – Approximately Maximum-Likelihood Trees for Large Alignments. *5*.

Quinlan, A.R., and Hall, I.M. (2010). BEDTools: a flexible suite of utilities for comparing genomic features. Bioinformatics *26*, 841–842.

Seemann, T. mlst. https://github.com/tseemann/mlst

Shen, W., and Xiong, J. (2019). TaxonKit: a cross-platform and efficient NCBI taxonomy toolkit. BioRxiv 513523.

Team, R.C. (2017). R: A language and environment for statiscal computing.

Team, Rs. (2020). RStudio: Integrated development for R.

Voorhies, A.A., Mark Ott, C., Mehta, S., Pierson, D.L., Crucian, B.E., Feiveson, A., Oubre, C.M., Torralba, M., Moncera, K., Zhang, Y., et al. (2019). Study of the impact of long-duration space missions at the International Space Station on the astronaut microbiome. Sci. Rep. *9*, 1–17.

Wickham, H., Averick, M., Bryan, J., Chang, W., McGowan, L., François, R., Grolemund, G., Hayes, A., Henry, L., Hester, J., et al. (2019). Welcome to the Tidyverse. J. Open Source Softw. *4*, 1686.
